# Supplementary material for: Nodal immune flare mimics nodal disease progression following neoadjuvant immune checkpoint inhibitors in non-small cell lung cancer
Source: Nat Commun. 2021 Aug 19;12:5045. doi: 10.1038/s41467-021-25188-0 (PMC8376947; doi:10.1038/s41467-021-25188-0)
Supplement: Supplementary file 1 — Supplementary information file. [file 41467_2021_25188_MOESM1_ESM.pdf]

Supplementary Information

Nodal Immune Flare Mimics Nodal Disease Progression Following Neoadjuvant Immune Checkpoint Inhibitors in Non-Small Cell Lung Cancer

Tina Cascone[1,*], Annikka Weissferdt[2,3], Myrna C. B. Godoy[4], William N. William Jr.[1,5], Cheuk H. Leung[6], Heather Y. Lin[6], Sreyashi Basu[7], Shalini S. Yadav[7], Apar Pataer[3], Kyle G. Mitchell[3], Md Abdul Wadud Khan[8], Yushu Shi[6,9], Cara Haymaker[10], Luisa M. Solis[10], Edwin R. Parra[10], Humam Kadara[10], Ignacio I. Wistuba[1,10], Padmanee Sharma[7,11], James P. Allison[7,12], Nadim J. Ajami[13], Jennifer A. Wargo[8], Robert R. Jenq[13,14], Don L. Gibbons[1], J. Jack Lee[6], Stephen G. Swisher[3], Ara A. Vaporciyan[3], John V. Heymach[1,*,#], Boris Sepesi[3,#]

Author Affiliations:

[1]Department of Thoracic/Head and Neck Medical Oncology, The University of Texas MD Anderson Cancer Center, 1515 Holcombe Blvd., Unit 432, Houston, TX, 77030.

[2]Department of Pathology, The University of Texas MD Anderson Cancer Center, 1515 Holcombe Blvd., Houston, TX, 77030.

[3]Department of Thoracic and Cardiovascular Surgery, The University of Texas MD Anderson Cancer Center, 1515 Holcombe Blvd., Houston, TX, 77030.

[4]Department of Thoracic Imaging, The University of Texas MD Anderson Cancer Center, 1515 Holcombe Blvd., Houston, TX, 77030.

[5]Hospital BP, a Beneficencia Portuguesa de Sao Paulo, R. Maestro Cardim 769, Sao Paulo, Brazil.

[6]Department of Biostatistics, The University of Texas MD Anderson Cancer Center, 1400 Pressler Street, Unit 1411, Houston, TX, 77030.

[7]The Immunotherapy Platform, The University of Texas MD Anderson Cancer Center, 1515 Holcombe Blvd., Houston, TX, 77030.

[8]Department of Surgical Oncology, The University of Texas MD Anderson Cancer Center, 1515 Holcombe Blvd., Houston, TX, 77030.

[9]Department of Statistics, The University of Missouri, Columbia, MO, 65201.

[10]Department of Translational Molecular Pathology, The University of Texas MD Anderson Cancer Center, 1515 Holcombe Blvd., Houston, TX, 77030.

[11]Department of Genitourinary Medical Oncology, The University of Texas MD Anderson Cancer Center, 1515 Holcombe Blvd., Houston, TX, 77030.

[12]Department of Immunology, The University of Texas MD Anderson Cancer Center, 1515 Holcombe Blvd., Houston, TX, 77030.

[13]Department of Genomic Medicine, The University of Texas MD Anderson Cancer Center, 1515 Holcombe Blvd., Houston, TX, 77030.

[14]CPRIT Scholar in Cancer Research, Houston, USA.

[#]These authors contributed equally.

*Corresponding authors.

Supplementary Figures and Legends

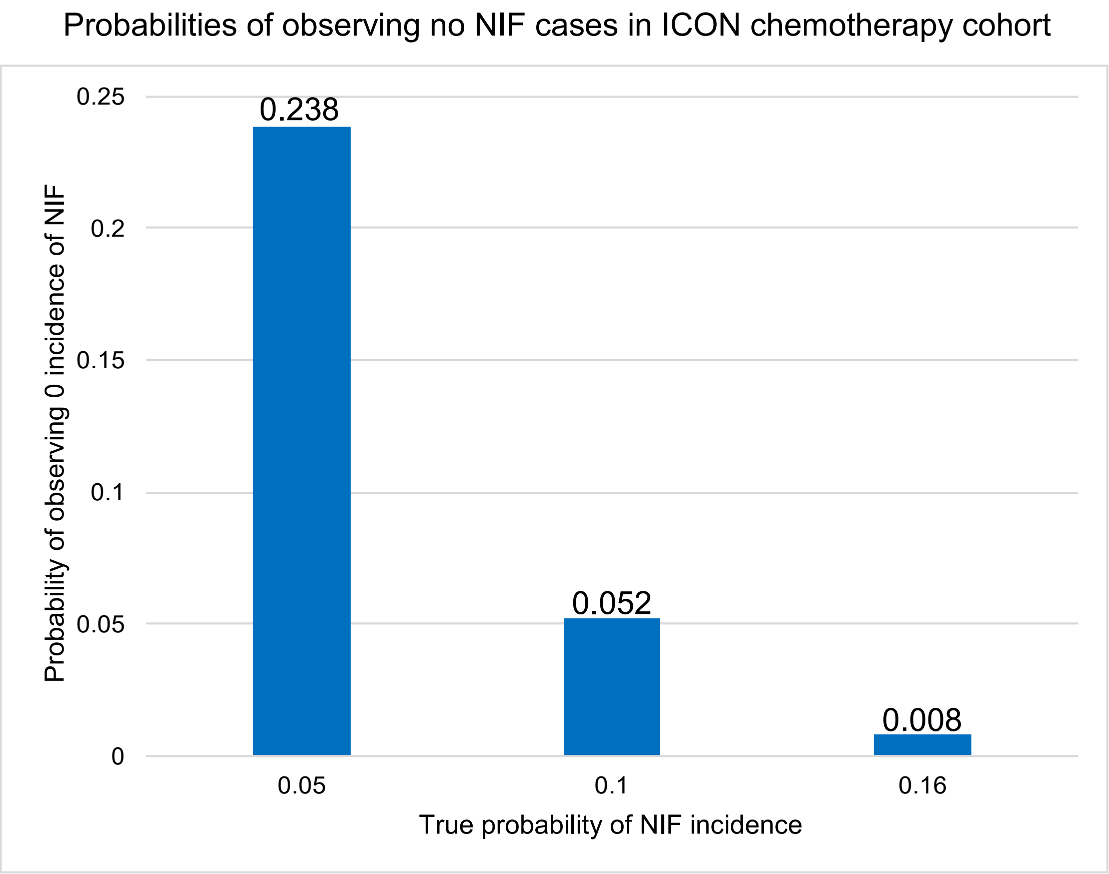

Supplementary Figure 1

Supplementary Figure 1. Probabilities of observing no NIF incidence in ICON cohort. The probabilities of observing 0 incidence of NIF in the 28 ICON patients were 23.8%, 5.2%, and only 0.8% if the true NIF rates were 5%, 10%, and 16%, respectively. The blue bars represent the probability of observing 0 incidence of NIF. The calculations were performed using the binomial distribution. ICON, ImmunogenomiC prOfiling in NSCLC; NIF, Nodal immune flare. Source data are provided as a Source Data file.

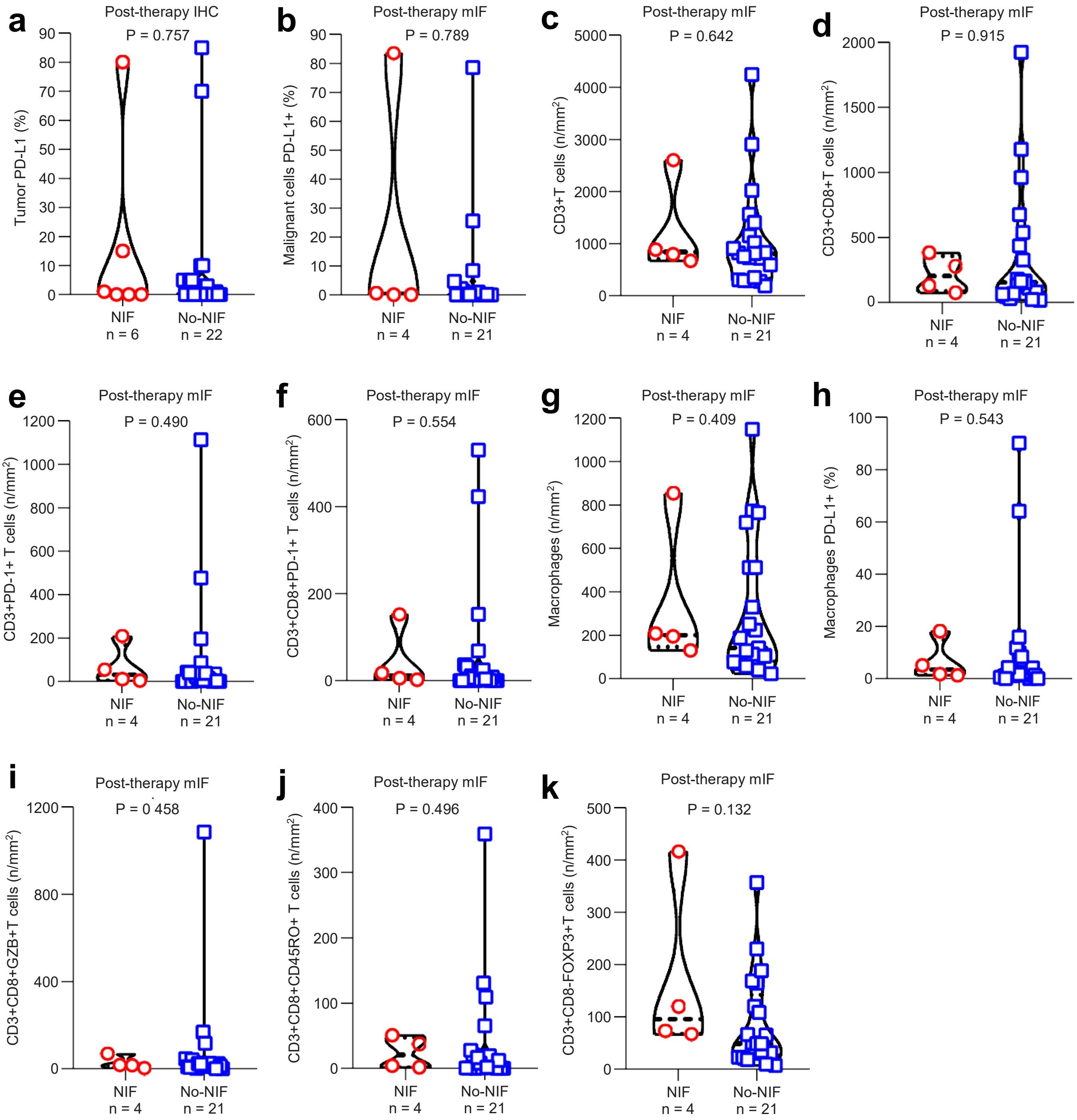

Supplementary Figure 2

Supplementary Figure 2. Immune infiltrates in post-therapy tumor samples of NEOSTAR patients with NIF/non-caseating granulomas and No-NIF. a, Post-therapy tumor PD-L1 (%) expression by immunohistochemistry (IHC) in malignant cells from NIF (n=6) and No-NIF (n=22) patients. b-h, Post-therapy mIF staining of tumor samples for cell populations identified with co-expression markers in panel 1 as (b) malignant cells PD-L1+ (%), (c) CD3+ T cells (n/mm^2), (d) CD3+CD8+ T cells (n/mm^2), (e) CD3+PD-1+ T cells (n/mm^2), (f) CD3+CD8+PD-1+ T cells (n/mm^2), (g) macrophages (n/mm^2), (h) macrophages PD-L1+ (%) from NIF (n=4) and No-NIF (n=21) patients. i-k, Post-therapy mIF staining of tumor samples for cell populations identified with co-expression markers in panel 2 as (i) CD3+CD8+GZB+ T cells (n/mm^2), (j) CD3+CD8+CD45RO+ T cells (n/mm^2), (k) CD3+CD8-FOXP3+ T cells (n/mm^2) from NIF (n=4) and No-NIF (n=21) patients. The NIF group for both IHC and mIF analyses included available tumor samples from patients with pathological evidence of non-caseating granulomas. Experiments and scorings/quantifications related to the presented results were conducted once. Data are presented as median with minima, lower and upper quartiles, and maxima. The dashed line indicates the median; the dotted lines indicate the lower quartile and upper quartile values; top and bottom of the violin plots indicate the maxima and minima. Two-sided P values are from Wilcoxon rank-sum test. The red circles depict data from the NIF group, and the blue squares depict data from the No-NIF group. IHC, immunohistochemistry; mIF, multiplex immunofluorescence. Source data are provided as a Source Data file.

Supplementary Tables

Supplementary Table 1. Proportion of patients with or without post-therapy tumor PD-L1 in No-NIF and NIF groups.

		No-NIF	NIF	P value
	Status	n (%)	n (%)	
Post-therapy tumor PD-L1	< 1%	12 (55)	3 (50)	1.00
	≥ 1%	10 (45)	3 (50)	

NIF, nodal immune flare; NIF group includes available post-therapy tissue samples with non-caseating granulomas. n, number of patients. Two-sided P value is from unconditional exact test with z-pooled statistic.

Supplementary Table 2. Immune cell gene expression scores by NanoString in resected nodes following neoadjuvant ICIs in NIF and No-NIF groups.

Immune cells		No-NIF		NIF	P value
	n	Expression score median (min, max)	n	Expression score median (min, max)	
CD45 cells	29	10.69 (9.91, 11.12)	8	10.92 (10.55, 12.28)	0.043
Macrophages	29	10.33 (9.66, 12.16)	8	11.01 (10.27, 13.14)	0.012
DCs	29	6.28 (4.9, 7.84)	8	7.61 (6.88, 8.24)	0.0001
Cytotoxic cells	29	8.30 (7.56, 9.00)	8	8.66 (8.19, 9.55)	0.086
Th1 cells	29	6.47 (4.71, 7.45)	8	6.76 (6.37, 7.13)	0.073
Exhausted CD8 T cells	29	5.87 (4.61, 6.96)	8	7.00 (6.00, 8.69)	0.003

Expression score is shown as median log_2 normalized count. NIF group includes available post-therapy samples with non-caseating granulomas. n, number of patients; DCs, dendritic cells; Th1, T helper 1 cells. Two-sided P value is from Wilcoxon rank-sum test. Source data are provided as a Source Data file.

Supplementary Table 3. Association of NIF with MPR in ITT population of NEOSTAR patients.

Pathologic response	Overall	NIF	No-NIF	P value
	n (%)	n (%)	n (%)	
pCR + MPR (0 - 10 % viable tumor)	13 (30)	1 (14)	12 (32)	0.357
No MPR, No surgery performed on trial	31(70)	6 (86)*	25 (68)	
Total	44 (100)	7 (100)	37 (100)	

NIF, nodal immune flare; NIF includes cases with radiologically abnormal nodes in absence of malignancy and with non-caseating granulomas on pathological evaluation. pCR, pathologic complete response; MPR, major pathologic response. n, number of patients. *Two patients underwent surgery off trial, one patient declined surgery. Two-sided P value is from unconditional exact test with z-pooled statistic.

Supplementary Table 4. Association of NIF with the percentage viable tumor cells in NEOSTAR patients resected on trial.

	NIF*		No-NIF^		P value
	n	Median (min, max)	n	Median (min, max)	
% viable tumor cells	4	38 (0, 62)	33	33 (0, 97.5)	0.842

NIF, nodal immune flare; NIF includes cases with radiologically abnormal nodes in absence of malignancy and with non-caseating granulomas on pathological evaluation. n, number of patients. *Two patients had surgery off trial and one patient declined surgery. The percentage of viable tumor for those resected off trial was not included. ^Four patients did not undergo surgery. Two-sided P value is from Wilcoxon rank-sum test. Source data are provided as a Source Data file.

Supplementary Table 5. Association of NIF with radiographic responses in NEOSTAR patients.

Radiographic response	Overall	NIF*	No-NIF	P value
	n (%)	n (%)	n (%)	
CR/PR	9 (21)	1 (14)	8 (22)	1.00
SD/PD, N/E	35 (79)	6 (86)	29 (78)	
Total	44 (100)	7 (100)	37 (100)	

NIF, nodal immune flare; NIF includes cases with radiologically abnormal nodes in absence of malignancy and with non-caseating granulomas on pathological evaluation. CR, complete response; PR, partial response; SD, stable disease; PD, progressive disease; n, number of patients. *One patient was not radiographically evaluable on trial as received one dose of ICIs complicated by treatment-related adverse event (TRAE) and underwent radiographic restaging after neoadjuvant chemotherapy off trial. N/E, not evaluable. Two-sided P value is from unconditional exact test with z-pooled statistic.

Supplementary Table 6. Association of treatment-related adverse events (TRAEs) by grade in NIF as compared to No-NIF patients in NEOSTAR study.

	TRAE Grade 1-2 n (%)	TRAE Grade 3-5 n (%)	P value
NIF	4 (67)	2 (33)	0.141
No-NIF	28 (90)	3 (10)	

NIF, nodal immune flare; NIF includes cases with radiologically abnormal nodes in absence of malignancy and with non-caseating granulomas on pathological evaluation. TRAE, treatment-related adverse event; n, number of patients. Seven of 44 patients did not have TRAE (1 NIF, 6 No-NIF). Two-sided P value is from unconditional exact test with z-pooled statistic.

Supplementary Table 7. Frequencies of treatment-related adverse events (TRAEs) in NIF patients of NEOSTAR study.

TRAE			NIF (n = 7)			
	Any grade		Grade 1-2		Grade 3-5	
	Count	%	Count	%	Count	%
Fatigue	4	57	4	57		
Rash acneiform	3	43	3	43		
Diarrhea	2	29	1	14	1	14
Anemia	2	29	2	29		
Nausea	2	29	2	29		
Hypermagnesemia	1	14	0	0	1	14
Alanine aminotransferase increased	1	14	1	14		
Blood bilirubin increased	1	14	1	14		
Headache	1	14	1	14		
Hyponatremia	1	14	1	14		
Psoriasis	1	14	1	14		
Hemoptysis	1	14	1	14		
Pruritus	1	14	1	14		
Vomiting	1	14	1	14		

NIF, nodal immune flare; NIF includes cases with radiologically abnormal nodes in absence of malignancy and with non-caseating granulomas on pathological evaluation. TRAE, treatment-related adverse event; NIF, nodal immune flare. n, number of patients. One patient did not have TRAE.

Supplementary Table 8. Frequencies of treatment-related adverse events (TRAEs) in No-NIF patients of NEOSTAR study.

TRAE			No-NIF (n = 37)			
	Any grade		Grade 1-2		Grade 3-5	
	Count	%	Count	%	Count	%
Rash acneiform	14	38	14	38		
Fatigue	11	30	11	30		
Cough	7	19	7	19		
Diarrhea	7	19	7	19		
Nausea	6	16	6	16		
Hyponatremia	4	11	3	8	1	3
Anemia	3	8	3	8		
Dyspnea	3	8	3	8		
Pruritus	3	8	3	8		
Pneumonitis	2	5	1	3	1	3
Alanine aminotransferase increased	2	5	2	5		
Chills	2	5	2	5		
Flu-like symptoms	2	5	2	5		
Headache	2	5	2	5		
Hyperthyroidism	2	5	2	5		
Hypomagnesemia	2	5	2	5		
Hypothyroidism	2	5	2	5		
Myalgia	2	5	2	5		
Sinus tachycardia	2	5	2	5		
WBC increased	2	5	2	5		
Hypoxia	1	3	0	0	1	3
Pneumonia	1	3	0	0	1	3
ANC increased	1	3	1	3		
Anorexia	1	3	1	3		
Aspartate aminotransferase increased	1	3	1	3		
Atypical Guillain-Barre` syndrome	1	3	1	3		
Blurred vision	1	3	1	3		
BUN increased	1	3	1	3		
Creatinine increased	1	3	1	3		
Dry skin	1	3	1	3		
Eye pain	1	3	1	3		
Fever	1	3	1	3		
Generalized muscle weakness	1	3	1	3		
Hemoptysis	1	3	1	3		

Hypernatremia	1	3	1	3			
Hyperuricemia	1	3	1	3			
Hypotension	1	3	1	3			
Myositis	1	3	1	3			
Non-cardiac chest pain	1	3	1	3			
Pain	1	3	1	3			
Rash maculopapular	1	3	1	3			
Urticaria	1	3	1	3			
Vomiting	1	3	1	3			

NIF, nodal immune flare; NIF includes cases with radiologically abnormal nodes in absence of malignancy and with non-caseating granulomas on pathological evaluation. WBC, White blood count; ANC, absolute neutrophil count; BUN; blood urea nitrogen. n, number of patients. Six patients did not have TRAE.
