# Supplementary material for: Nodal immune flare mimics nodal disease progression following neoadjuvant immune checkpoint inhibitors in non-small cell lung cancer
Source: Nat Commun. 2021 Aug 19;12:5045. doi: 10.1038/s41467-021-25188-0 (PMC8376947; doi:10.1038/s41467-021-25188-0)
Supplement: Supplementary file 2 — Description of Additional Supplementary Files. [file 41467_2021_25188_MOESM2_ESM.pdf]

Title: Supplementary Data 1.
Description: NanoString dataset of log2 normalized counts in NIF and No-NIF nodes resected after neoadjuvant ICIs in the NEOSTAR randomized study
