# Supplementary material for: Nodal immune flare mimics nodal disease progression following neoadjuvant immune checkpoint inhibitors in non-small cell lung cancer
Source: Nat Commun. 2021 Aug 19;12:5045. doi: 10.1038/s41467-021-25188-0 (PMC8376947; doi:10.1038/s41467-021-25188-0)
Supplement: Supplementary file 4 — Reporting summary. [file 41467_2021_25188_MOESM4_ESM.pdf]

nature research

Corresponding author(s): Tina Cascone, MD PhD and John V. Heymach, MD, PhD

Last updated by author(s): Jul 22, 2021

Reporting Summary

Nature Research wishes to improve the reproducibility of the work that we publish. This form provides structure for consistency and transparency in reporting. For further information on Nature Research policies, see our Editorial Policies and the Editorial Policy Checklist.

Statistics

For all statistical analyses, confirm that the following items are present in the figure legend, table legend, main text, or Methods section.

n/a	Confirmed	
☐	☒	The exact sample size (*n*) for each experimental group/condition, given as a discrete number and unit of measurement
☐	☒	A statement on whether measurements were taken from distinct samples or whether the same sample was measured repeatedly
☐	☒	The statistical test(s) used AND whether they are one- or two-sided<br>*Only common tests should be described solely by name; describe more complex techniques in the Methods section.*
☒	☐	A description of all covariates tested
☐	☒	A description of any assumptions or corrections, such as tests of normality and adjustment for multiple comparisons
☐	☒	A full description of the statistical parameters including central tendency (e.g. means) or other basic estimates (e.g. regression coefficient) AND variation (e.g. standard deviation) or associated estimates of uncertainty (e.g. confidence intervals)
☐	☒	For null hypothesis testing, the test statistic (e.g. *F*, *t*, *r*) with confidence intervals, effect sizes, degrees of freedom and *P* value noted<br>*Give P values as exact values whenever suitable.*
☐	☒	For Bayesian analysis, information on the choice of priors and Markov chain Monte Carlo settings
☒	☐	For hierarchical and complex designs, identification of the appropriate level for tests and full reporting of outcomes
☐	☒	Estimates of effect sizes (e.g. Cohen's *d*, Pearson's *r*), indicating how they were calculated

Our web collection on statistics for biologists contains articles on many of the points above.

Software and code

Policy information about availability of computer code

Data collection

Clinical data were collected via the Data Management Initiative (DMI) project at the University of Texas MD Anderson Cancer Center and Microsoft Excel (v. 2016) Spreadsheets.

Radiological data: CT scans were acquired in a multidetector scanner following IV contrast administration unless contra-indicated. Multiplanar CT image series were reconstructed with 2.5 mm slice thickness using standard and high spatial reconstruction algorithms. FDG-PET/CT imaging was performed using Discovery STE PET/CT scanner (GE healthcare Waukesha WI). All patients fasted for 6 hours before the FDG injection and had confirmed normal fasting blood glucose level of less than 200 mg/dL. PET was performed in 3-dimensional mode at 3 – 5 min per bed station depending on patient BMI. An intravenous injection of 9-11 mCi of FDG was administered in the arm or central venous catheter on the side opposite to the cancer, and emission scans were acquired at 70 ± 10 minutes after the FDG injection. The acquired PET data were corrected for scatter coincidences, random coincidences, deadtime, and attenuation and reconstructed using OSEM on standard vendor-provided workstations. Non-contrast-enhanced CT images from the base of the skull to the mid-thigh were acquired in helical mode (speed, 13.5 mm per rotation) during shallow breathing at a 3.75-mm slice thickness, a tube voltage of 120 kVp, and 0.5-second rotation with tube current modulation. Daily quality control procedures were performed on all PET scanners to ensure cross-calibration between systems and normalize differences in system performance. In a small number of patients, CT or PET/CT scans were performed at outside institution with comparable technique.

Pathological data collection was performed with routine cytophatological/histopathological processing of tissue samples as detailed in the Methods session "Pathological analysis" of the manuscript.

Immunohistochemistry (IHC) of PD-L1 data: Available tissue samples post neoadjuvant immunotherapy for PD-L1 staining were collected from patients on NEOSTAR trial. Data were collected using Microsoft Excel (v. 2016) Spreadsheets.

Multiplex immunofluorescence (mIF) staining data: Available tissue samples post neoadjuvant immunotherapy for mIF staining were collected

from patients on NEOSTAR trial. Stained slides were scanned using the multispectral microscope, Vectra 3.0.3 imaging system (Akoya Biosciences/PerkinElmer). The phenochart 1.0.9 viewer (Akoya Biosciences/PerkinElmer) was employed to select ROIs in each sample using the InForm 2.8.2 image analysis software (Akoya Biosciences/PerkinElmer). Data were consolidated using the R studio 3.5.3 (Phenopter 0.2.2 packet, Akoya Biosciences/PerkinElmer) and SAS 7.1 Enterprise. Data were collected using Microsoft Excel v.2016 and plotted using GraphPad prism v.8.0.0.

NanoString and GSEA data: Available nodal samples were collected post neoadjuvant immunotherapy on NEOSTAR trial. Total RNA was extracted using the RecoverALL™ Total Nucleic Acid Isolation kit (Ambion, Austin, TX) according to the manufacturer's instructions. RNA quality and quantity were assessed using the Nanodrop spectrometer (ND-Nanodrop1000, Thermo Scientific, Wilmington, MA, USA). For the assay, 100 ng of RNA was used to detect immune gene expression using the nCounter PanCancer Immune Profiling panel along with custom CodeSet.

Gut microbiome data: Fecal samples were collected from patients on NEOSTAR trial as previously reported (https://pubmed.ncbi.nlm.nih.gov/33603241/). Total DNA was extracted from fecal samples using the QIAamp DNA Stool Kit (Qiagen, Hilden, Germany), including a bead-beating lysis step. VSEARCH v2.10.4 was used to merge and de-replicate paired-end reads and sorted them by length and size. Sequences were then error-corrected and chimera-filtered using the UNOISE algorithm v.3 and generated Operational Taxonomic Units (OTUs) and presumed chimeras.

Data analysis

Radiological data: Tumor volume measurement was performed using a commercially available semi-automatic software MIM v. 6.6.6 (MIM Software Inc., Cleveland, Ohio). Characterization of nodal size on CT was performed by measuring short axis diameter using mediastinal window setting (level = 50; width = 350). Characterization of tumor and lymph node 18F-FDG uptake was performed using semiquantitative analysis of the SUVmax (MIM version 6.6.6; MIM Software Inc., Cleveland, Ohio).

Linear mixed-effect model was used to consider the intra-individual correlation of multiple lymph nodes for the nodal size and nodal SUVmax analyses in ICON and NEOSTAR cohorts and for the tumor volume and tumor SUVmax analyses in the NEOSTAR cohort. Unconditional exact test available in the R package "Exact" was used to compare the categorical distributions between patient populations. Analyses were performed in R 3.6.3 and UNIVARIATE, NPAR1WAY, and MIXED procedures in SAS 9.4

Tissue IHC and mIF staining data analyses were performed as previously reported (https://pubmed.ncbi.nlm.nih.gov/33603241/) and as detailed in the Methods section of the manuscript.

NanoString and Gene Set Enrichment Analysis (GSEA): nCounter Digital Analyzer was used to tabulate the counts of the reporter probes and for further analysis raw data output was imported into nSolver (http://www.nanostring.com/products/nSolver). Normalization, cell type and differential gene expression analyses were performed using the nSolver Advanced data analysis package. Gene set enrichment analysis (GSEA) was performed using GSEA software (https://www.gsea-msigdb.org). The data were collected using Microsoft Excel v.2016 and plotted using GraphPad prism v. 8.0.0.

Gut Microbiome: The V4 region of bacterial 16S ribosomal-RNA V4 region was amplified and sequenced on the Illumina MiSeq platform with 2 x 250 bp reads (Illumina, Inc, San Diego, CA). We used VSEARCH v2.10.4 to merge and de-replicate paired-end reads and sorted them by length and size. Sequences were then error-corrected and chimera-filtered using the UNOISE algorithm v. 3 and generated Operational Taxonomic Units (OTUs) and presumed chimeras. Later, we added the chimeras sequences identified by the UNOISE algorithm v.3 but matched an entry in Silva database v. 138 with a perfect score back to the OTU list and generated a total of 1,849 OTUs. The sequencing depths ranged from 1,339 to 175,238, with a median of 11,656 reads per sample. Alpha diversity was calculated using Inverse Simpson Index. Weighted-unifrac dissimilarity indices were used to calculate the pairwise dissimilarities and perform principal coordinate analysis (PCoA) between samples. Alpha and beta diversities were calculated using QIIME 1.9.0.32. Linear Discriminant Analysis (LDA) was performed using the LDA Effect Size (LEfSe) algorithm for comparing bacterial taxa at the genus level between groups. LDA score of 2 and two-sided P value of 0.05 were used as cutoff. Two-sided P values were from Mann Whitney U test. The results were plotted in R (R Core Team 2020; https://www.R-project.org) using ggplot2 package (https://ggplot2.tidyverse.org) and GraphPad Prism v.8.00.

For manuscripts utilizing custom algorithms or software that are central to the research but not yet described in published literature, software must be made available to editors and reviewers. We strongly encourage code deposition in a community repository (e.g. GitHub). See the Nature Research guidelines for submitting code & software for further information.

Data

Policy information about availability of data

All manuscripts must include a data availability statement. This statement should provide the following information, where applicable:

- Accession codes, unique identifiers, or web links for publicly available datasets
- A list of figures that have associated raw data
- A description of any restrictions on data availability

The authors declare that the data supporting the findings of this study are available within the manuscript, its supplementary information files and the Source Data. The 16S fecal microbiome sequencing data (supporting the findings in Fig. 5) are publicly available in the National Center for Biotechnology Information Sequence Read Archive (SRA BioProject ID PRJNA665109; https://www.ncbi.nlm.nih.gov/bioproject/665109). Taxonomy was assigned using the Silva database (https://www.arb-silva.de/) for 16S rRNA sequences. The NanoString log2 normalized counts data that support the findings of this study (Fig. 4 and Supplementary Table 2) are available as Supplementary Data with the manuscript. Other relevant de-identified data/information related to the current study that can be shared will be available from the corresponding authors (T.C. and J.V.H.) within a reasonable timeframe and upon reasonable academic request and will require the researcher to sign a data access agreement with the University of Texas MD Anderson Cancer Center as the information includes data collected under an institutional alliance clinical trial and/or an institutional observational protocol.

Field-specific reporting

Life sciences study design

All studies must disclose on these points even when the disclosure is negative.

Sample size	No specific sample size calculations were performed for this study, as this study focuses on a secondary analysis of a randomized phase 2 trial (NEOSTAR) from which primary and select secondary and exploratory outcomes have been previously reported (https://pubmed.ncbi.nlm.nih.gov/33603241/). Forty-four patients included in the NEOSTAR trial (intention-to-treat population) were included in the present study. For the patients included in this study from the ICON project, no sample size calculations were performed. The ICON project enrolled patients at our institution with stage IB-IIIA non-small cell lung cancer who underwent surgical resection. Twenty-eight patients enrolled on the ICON project who received neoadjuvant chemotherapy were included in the current study.
Data exclusions	Data for all patients with available datapoints were analyzed; there were no additional exclusions.
Replication	In the present study, experiments and scoring/quantification related to the reported results were performed once. Replication was not performed as the analyzed radiological images and specimens were unique patient imaging scans and samples. All techniques and reagents used for the correlative analyses of this study had been previously optimized and validated.
Randomization	In the randomized NEOSTAR trial, after confirmation of eligibility criteria, patients were stratified by stage (I vs. II vs. III) and randomized at 1:1 ratio to either nivolumab alone or the combination of nivolumab and ipilimumab using a minimization technique. The results presented in the current manuscript are based on novel clinical observations that occurred as the NEOSTAR trial was progressing and on retrospective analyses of the ICON study. All analyses related to the current study were initially unplanned as the NIF phenomenon was not anticipated as part of the trial and is the result of an ad hoc analysis of the NEOSTAR clinical trial. No randomization was performed as part of the ICON observational study.
Blinding	The NEOSTAR trial was not a blinded study. However, the pathological response evaluation after neoadjuvant therapy was reviewed in a blinded manner by two pathologists. For the current manuscript reporting on the NIF phenomenon, the radiologist who assessed radiological nodal findings was blinded to the pathological nodal findings of non-caseating granulomas, and the pathologist who evaluated the pathological nodal findings of non-caseating granulomas was blinded to the radiological nodal findings. The investigators involved in the tissue immune profiling determining PD-L1 IHC and mIF staining were blinded to the NIF status. The investigators involved in the nodal NanoString and gut microbiome analyses were not blinded to the NIF status as this information was needed to allocate the samples in each group (NIF vs. No-NIF) for the data analysis. Blinding was not possible for the ICON observational study.

Reporting for specific materials, systems and methods

We require information from authors about some types of materials, experimental systems and methods used in many studies. Here, indicate whether each material, system or method listed is relevant to your study. If you are not sure if a list item applies to your research, read the appropriate section before selecting a response.

Materials & experimental systems

n/a	Involved in the study	
☐	☒	Antibodies
☒	☐	Eukaryotic cell lines
☒	☐	Palaeontology and archaeology
☒	☐	Animals and other organisms
☐	☒	Human research participants
☐	☒	Clinical data
☒	☐	Dual use research of concern

Methods

n/a	Involved in the study	
☒	☐	ChIP-seq
☒	☐	Flow cytometry
☒	☐	MRI-based neuroimaging

Antibodies

Antibodies used	Immunohistochemistry (IHC) studies for PD-L1 staining in malignant cells: PD-L1 anti-human antibody clone 28-8, dilution 1:100; Abcam cat# ab205921, Cambridge, MA, USA.<br><br>Multiplex Immunofluorescence studies: antibodies against the following markers were used, Panel 1: cytokeratin (clone AE1/AE3, cat# M351501-2, dilution 1:300, Dako, Santa Clara, CA), PD-L1 (clone E1L3N, cat# 13684S, dilution 1:3000, Cell Signaling Technology, Danvers, MA), CD68 (clone PG-M1, cat# M087601-2, dilution 1:450, Dako), CD3 (polyclonal, cat# IS503, dilution 1:100, Dako), CD8 (clone C8/144B, cat# MS-457-S, dilution 1:300, Thermo Fisher Scientific, Waltham, MA), and PD-1 (clone EPR4877-2, cat# ab137132, dilution 1:250, Abcam, Cambridge, MA); and Panel 2: cytokeratin (clone AE1/AE3, cat# M351501-2, dilution 1:300, Dako), CD3 (polyclonal, cat# IS503, dilution 1:100, Dako), CD8 (clone C8/144B, cat# MS-457-S, dilution 1:300, Thermo Fisher Scientific), CD45RO (clone UCHL1, cat# PA0146, Cell Signaling Technology), Granzyme B (clone 11F1, cat# PA0291,Cell Signaling Technology), and FOXP3 (clone D2W8E, cat# 98377S, dilution 1:50, Cell Signaling Technology). All markers were sequentially applied and stained using their respective fluorophores in the Opal 7 kit (catalogue #NEL797001KT; Akoya Biosciences/PerkinElmer, Waltham, MA).
Validation	Immunohistochemistry (IHC) antibody for PD-L1 (PD-L1 anti-human antibody clone 28-8, dilution 1:100; Abcam cat# ab205921, Cambridge, MA, USA) staining in malignant cells was validated as reported in https://www.ncbi.nlm.nih.gov/pubmed/28719380.

Multiplex Immunofluorescence antibodies against the following markers, Panel 1: cytokeratin (clone AE1/AE3, cat# M351501-2, dilution 1:300, Dako, Santa Clara, CA), PD-L1 (clone E1L3N, cat# 13684S, dilution 1:3000, Cell Signaling Technology, Danvers, MA), CD68 (clone PG-M1, cat# M087601-2, dilution 1:450, Dako), CD3 (polyclonal, cat# IS503, dilution 1:100, Dako), CD8 (clone C8/144B, cat# MS-457-S, dilution 1:300, Thermo Fisher Scientific, Waltham, MA), and PD-1 (clone EPR4877-2, cat# ab137132, dilution 1:250, Abcam, Cambridge, MA); and Panel 2: cytokeratin (clone AE1/AE3, cat# M351501-2, dilution 1:300, Dako), CD3 (polyclonal, cat# IS503, dilution 1:100, Dako), CD8 (clone C8/144B, cat# MS-457-S, dilution 1:300, Thermo Fisher Scientific), CD45RO (clone UCHL1, cat# PA0146, Cell Signaling Technology), Granzyme B (clone 11F1, cat# PA0291,Cell Signaling Technology), and FOXP3 (clone D2W8E, cat# 98377S, dilution 1:50, Cell Signaling Technology) were validated as reported in https://pubmed.ncbi.nlm.nih.gov/29042640.

Human research participants

Policy information about studies involving human research participants

Population characteristics	The randomized NEOSTAR trial enrolled 44 patients with stage I-IIIA (N2 single station) operable non-small cell lung cancer. A full description of population characteristics has been reported (https://pubmed.ncbi.nlm.nih.gov/33603241/) and is also summarized in Table 1 of the current study.<br><br>The ICON project at our Institution enrolled patients with stage IB-IIIA non-small cell lung cancer who underwent surgical resection. Of the patients enrolled in the ICON project, 28 patients who received neoadjuvant chemotherapy were included in the present study. A full description of the ICON patient population characteristics is provided in Table 1 of the current study.
Recruitment	Patients with resectable stage IB-IIIA non-small cell lung cancer who were treated at our institution and underwent surgical resection were recruited for the ICON study. The ICON patients included in the current study received neoadjuvant chemotherapy. The voluntary nature of enrollment may have introduced a potential for self-selection bias in this patient cohort.<br><br>The NEOSTAR randomized study recruited patients with operable stage I-IIIA (N2 single station) at our institution. Patients who met eligibility criteria of the study were recruited, enrolled and treated in the Departments of Thoracic/Head and Neck Medical Oncology and Thoracic Surgery at The University of Texas MD Anderson Cancer Center. Potential biases applicable to this study were the relatively subjective disease resectability/operability prior to enrollment on study and the self-selection bias (derived potentially from patient health literacy regarding the trial). The impact of both these potential biases on the study results were minimized by presentation and discussion of eligible patients for the study at multidisciplinary tumor board conference prior to enrollment and by objectively evaluating the study endpoints. The impact of these potential biases on the results of the current manuscript are negligible as this work reports the results of an ad hoc analysis of the randomized NEOSTAR study.
Ethics oversight	The study was approved by the University of Texas MD Anderson Cancer Center's Institutional Review Board (NEOSTAR, NCT03158129; ImmunogeniC prOfiling in Non-small cell lung cancer, ICON, PA15-1112; Immunotherapy Platform umbrella protocol PA13-0291). This study complied with all relevant regulations regarding the use of human study participants and was conducted in accordance to the criteria set by the Declaration of Helsinki.

Note that full information on the approval of the study protocol must also be provided in the manuscript.

Clinical data

Policy information about clinical studies

All manuscripts should comply with the ICMJEguidelines for publication of clinical research and a completedCONSORT checklist must be included with all submissions.

Clinical trial registration	The NEOSTAR randomized trial is registered with ClinicalTrials.gov (NCT03158129). The ICON study is a noninterventional, observational study. The ICON study was approved by the University of Texas MD Anderson's Institutional Review Board (PA15-1112) and is not registered on ClinicalTrials.gov given its noninterventional, observational nature.
Study protocol	Information regarding the randomized NEOSTAR study protocol is available at ClinicalTrials.gov (NCT03158129).
Data collection	The randomized NEOSTAR trial enrolled patients at University of Texas MD Anderson Cancer Center in Houston, Texas. Patient enrollment started on June 16, 2017 and ended on November 15, 2018. The ICON study enrolled patients between 2016 and 2018 at the University of Texas MD Anderson Cancer Center in Houston, Texas. Electronic clinical report forms were collected through the Data Management Initiative (DMI) project at the University of Texas MD Anderson Cancer Center and Microsoft Excel (v. 2016) Spreadsheets.
Outcomes	The primary outcome for the NEOSTAR randomized trial is the major pathological response in patients treated with the study intervention/s (ClinicalTrials.gov, NCT03158129). The primary and select secondary and exploratory outcomes of the NEOSTAR randomized trial have been previously reported (https://pubmed.ncbi.nlm.nih.gov/33603241/). The present study represents a secondary analysis of patients on the randomized NEOSTAR trial focusing on the observation of nodal immune flare (NIF) phenomenon following neoadjuvant immune checkpoint inhibitor therapy. This analysis was not planned at the conception of the trial, since the NIF phenomenon has not been previously comprehensively evaluated and reported in NSCLC patients after neoadjuvant immunotherapies. NIF was observed throughout the randomized NEOSTAR trial. We report this analysis of NIF as an unplanned but clinically important and relevant analysis of the randomized NEOSTAR trial. No primary or secondary outcomes were specified for the ICON project, as it is an observational immunoprofiling project.
